# Illusory Temporal Binding in Meditators

**DOI:** 10.1007/s12671-016-0583-z

**Published:** 2016-08-02

**Authors:** Peter Lush, Jim Parkinson, Zoltan Dienes

**Affiliations:** 1 School of Psychology, University of Sussex and Sackler Centre for Consciousness Science, Brighton, UK; 2 School of Psychology, University of Sussex, Pevensey Building, Falmer, BN1 9QH UK

**Keywords:** Mindfulness, Intentions, Sense of agency, Time perception, Causal binding, Intentional binding

## Abstract

We investigate conditions in which more accurate metacognition may lead to greater susceptibility to illusion and thus conditions under which mindfulness meditation may lead to less accurate perceptions. Specifically, greater awareness of intentions may lead to an illusory compression of time between a voluntary action and its outcome (“intentional binding”). Here, we report that experienced Buddhist mindfulness meditators rather than non-meditators display a greater illusory shift of the timing of an outcome toward an intentional action. Mindfulness meditation involves awareness of causal connections between different mental states, including intentions. We argue that this supports improvements in metacognition targeted at motor intentions. Changes in metacognitive ability may result in an earlier and less veridical experience of the timing of action outcomes either through increased access to sensorimotor pre-representations of an action outcome or by affording greater precision to action timing judgements. Furthermore, as intentional binding is an implicit measure of the sense of agency; these results also provide evidence that mindfulness meditators experience a stronger sense of agency.

## Introduction

Mindfulness is a concept central to Buddhist traditions and can be defined as the cultivation of a calm (hence “non-judgemental”) awareness of present states, especially mental states, and specifically including an awareness of the temporal properties of the ongoing states, such as their transience (Williams and Kabat-Zinn 2011). While mindfulness practice often starts with awareness of the experiences of breathing, the central mindfulness practice of the Pali canon, Satipatthana, includes cultivating awareness of all mental states, including intentions and their consequences (Analayo 2004; Gunaratana 2012). Thus Dreyfus (2011) argued that mindfulness meditation should lead to cognitive differences in the awareness of intentions and urges psychologists to explore this possibility. Consistently, mindfulness meditators have been found to report earlier judgements of the timing of an intention to move than non-meditators, suggesting they have more accurate metacognition related to intention (Dienes et al. 2016; Lush Naish and Dienes 2016).

The sense of agency can be defined as the sense of being in control of one’s goal-directed actions and their outcomes in the world (Gallagher and Zahavi 2008; Haggard and Chambon 2012). The modern study of agency can be traced back to the work of Libet Gleason Wright and Pearl (1983), in which the timing of a conscious intention to move (W) was related to the “readiness potential” in the motor cortex which precedes voluntary motor actions. Libet et al. measured timing judgements by asking participants to watch a rotating “clock” and to retrospectively report the position of the clock “hand” had occupied when they experienced the urge to move. Intentional binding refers to the subjectively reported time compression that occurs between an intentional action and its outcome when compared to the timing of an action alone and of an event that does not depend upon an action (Haggard, Clark and Kalogeras 2002). It can be measured using Libet et al.’s rotating clock method to record the judged time of an action or an auditory tone in two conditions: a contingent condition in which the action causes the tone; and a baseline condition in which the action does not result in a tone or the tone occurs without an action. The effect emerges from the perceived shift of the action toward the tone (action binding) and of the tone toward the action that caused it (outcome binding) in the contingent condition compared to the baseline condition (Moore and Obhi 2012).

Intentional binding has been employed to investigate disorders that involve changes in the sense of agency e.g. in schizophrenia (Haggard Martin Taylor-Clarke Jeannerod and Franck 2003; Voss et al. 2010), alien limb syndrome (Wolpe et al. 2013) and functional motor disorder (Kranick et al. 2013) and in pharmacologically induced changes in sense of agency e.g. ketamine (Moore et al. 2011). There is, however, some evidence that weaker action-effect interval binding can occur in the absence of intention (Buehner 2012; Wohlschlager Haggard Gesierich and Prinz 2003). Therefore, intentional binding may be a special case of a more general causal binding of actions to their outcomes, with information specific to intentions supporting stronger binding.

There is not yet consensus on the mechanism of intentional binding. The important point is that the proposed mechanisms agree on intentional binding arising from metacognitive processes. Greater awareness of intentions should increase the binding effect. According to one theory, outcome binding arises from accurate prediction of action outcomes (Waszak, Cardoso-Leite and Hughes 2012). That is, the better one can predict the outcome from the intention, the sooner in time the outcome is perceived as happening. On other theories, the assessed time of each component is affected by a type of anchoring produced by the other event happening. In this case, the greater precision with which an intention and, hence, an intentional action is timed, the more it will affect the perceived time of the outcome (Kawabe Roseboom and Nishida 2013; Moore and Fletcher 2012). With regard to intentional binding being a measure of sense of agency, the more precisely one represents the outcomes of an intention as intentional outcomes, the more sense one has of acting on an intention; or the more precision with which one represents the intention, the more sense one has as acting on an intention. Thus, on each of these theories, greater awareness of intentions should lead to greater outcome binding, associated with a greater sense of agency.

Because Buddhist mindfulness involves awareness of “dependent origination” i.e. the causal connections between different mental states, including intentions and their outcomes (for example, in mindfulness of movements, Gunaratama 2012), meditators should have greater metacognitive access to their intentions. Meditation is an exercise in metacognitive processes, both in monitoring and control (Jankowski and Holas 2014) and sustained meditation practice seems likely to lead to changes in metacognition (Dienes et al. 2016). Consistently, meditators have been found to show more accurate metacognition related to the timing of an intention than non-meditators, (Lush Naish and Dienes 2016) and experienced meditators have been shown to have greater metacognitive access to negative deflections of slow cortical potentials (which, when averaged, produce the early readiness potential) (Jo Hinterberger Wittmann and Schmidt 2015).

Greater metacognitive accuracy of higher order mental states targeted at motor intentions should influence timing judgements that are dependent on prediction, such as outcome binding. These considerations can be used to make different predictions. On the one hand, meditators may be more accurate in their timing estimates due to improvements in metacognition and attention resulting from mindfulness practice (Jo et al. 2014). On the other hand, the ability to develop accurate prior expectations concerning the outcome of intentions, and to consistently and reliably use those expectations, could lead to perception of the tone as occurring early. In terms of this mechanism, this could result either from more reliable sensorimotor pre-representations of the outcome tone (Waszak et al. 2012) or through greater precision (and therefore greater weight) being afforded to intentional action in an outcome timing judgement (Wolpe et al. 2013). As there is some agreement that action binding is a result of cue integration, action binding would be smaller in meditators if there is a relative increase in precision of action judgements, reducing the relative contribution of the tone to action timing judgements (Wolpe and Rowe 2014).

Jo et al. (2014), the one previous study that has addressed the question of intentional binding in meditators, found no significant difference between meditators and non-meditators in intentional binding. However, a non-significant result does not in itself mean there is no effect. To assess the sensitivity of the study to pick up an effect, an estimate is needed of the sort of difference in intentional binding that could be found between different groups. Kranick et al. (2013) provided such an estimate using functional motor disorder patients; the difference between groups was on the order of magnitude of about half the effect found in control participants. Based on this estimate, Jo et al’s data do not provide evidence for there being no difference in intentional binding between meditators and non-meditators (see Supplemental materials).

We tested mindfulness meditators and age-matched controls on an intentional binding task. If mindfulness increases the relative precision afforded to action timing judgements, then the meditation group should show decreased action binding and increased outcome binding due to improved metacognitive access to motor intentions resulting from a history of sustained attention to intentions and actions. Alternatively, if meditators are more accurate in their timing estimates as a result of improved attentional abilities generally, their reported judgements should be more veridical and they should show less binding of any sort (Jo Hinterberger and Schmidt 2014).

## Method

### Participants

Eight meditators were recruited from Brighton-based Buddhist organisations. Eight age and gender matched controls were recruited by advertisement (3 males and 5 females, mean age = 49.3 years, *SD* = 10.5). The Buddhist meditators (3 males and 5 females, mean age = 49.1 years, *SD* = 9.8) reported a mean of 14.6 years of continuous meditation experience (*SD* = 11.6) and a mean of 23.3 (*SD* = 12.0) hours per month meditation. Meditators were asked to provide details of their practice in simple terms and all reported using a form of mindfulness meditation. All members of the control group reported that they had no experience of mindfulness meditation.

Ethical approval was received from the University of Sussex ethical committee. Informed consent was obtained from each participant before commencing with the study. Participants received cash payment of £5, and were additionally paid £5 in travel expenses.

Participants were recruited for the duration of one term, until there were no more responses. Bayesian analyses were used to assess sensitivity. As stopping was not conditional on the *p* value, orthodox statistics assuming fixed N could be used (Cox and Mayo 2010, section 10.1). Crucially, we used Bayesian analyses to indicate the strength of evidence for H1 versus H0; the measure of evidence is valid no matter what the stopping rule.

### Procedure

Visual stimuli were displayed at 100 Hz on a 21-in. CRT monitor and auditory stimuli were presented via Sennheiser headphones. For each trial, a clock face was presented, marked at thirty degree intervals and subtended a visual angle of five degrees. A static dot, subtending at 0.2°, appeared at a pseudo-randomised position and began rotating around the clock 250 ms later (at 2560 ms per revolution). Participants were seated at a viewing distance of approximately 60 cm. A computer keyboard was used to record actions (button presses).

There were four trial types, presented in separate blocks. In contingent trials, pressing a key triggered a 1000 Hz, 100 ms duration tone after a 250 ms delay. Participants were asked to look at a fixation cross in the centre of the clock and to wait for at least one revolution before pressing the button at a time of their choosing. The trial was restarted if the action occurred before one full revolution or after six revolutions. Participants were asked not to plan ahead or to aim for a particular point on the clock and to report either the action or the tone (to give contingent action or contingent tone judgements). Baseline action trials were the same as contingent action trials except the button did not trigger a tone. In baseline tone trials, the tone was triggered pseudo-randomly between 2.5 s and 7 s following one revolution of the clock.

Following the tone (or action on baseline action trials), the dot continued moving for a pseudo-randomised period of time between 1200 ms and 2370 ms. The clock was then removed from the screen for a pseudorandomised time interval (500 ms to 1280 ms). When the clock reappeared, participants were able to control the position of the dot with a mouse. Moving the mouse forward (toward the screen) caused the dot to move in a clockwise direction around the clock face and the reverse mouse movement (away from the screen) caused the dot to move counter-clockwise around the clock face. Participants were asked to move the dot to the position it had occupied at the time of the judged event (action or tone) and to press the mouse button to record their judgement.

Each block consisted of 40 repetitions of one trial type and blocks were separated by 30 s rest periods. The four blocks were presented in counterbalanced order. Before the session began, all participants were trained with four practice trials in the baseline tone condition and four in the baseline action condition so that they could become familiarised with the reporting procedure. All Stimuli were generated with Matlab running Psychtoolbox v3 (Kleiner et al. 2007).

### Measures

Mean judgement errors were calculated for each group on each trial type. Individual judgements more than 3.5 SD from the mean for each participant on each judgement type were excluded before mean judgement errors were calculated for each participant. Nine judgements were filtered by this method ((1.4 % of all trials). The adjusted mean errors for action and tone conditions were then subtracted from their respective contingent conditions to calculate action and outcome binding. Finally, outcome binding was subtracted from action binding to produce a total binding measure. We ran independent *t* tests to compare the two groups on these measures.

Within-participant SD of timing judgements provides a measure of precision in estimating the time of an event. If binding reflects the combination of cues according to the precision afforded to actions or their outcomes, any differences in intentional binding should be accompanied by differences in this measure. In terms of cue integration theory, it is the interaction between meditators vs controls by tone vs action timing precision that determines changes in intentional binding between groups. If meditators relative to controls have greater precision for actions rather than outcomes, then outcome binding should be greater and action binding less for meditators relative to controls.

### Data Analyses

Bayes factors (*B*) were used to assess strength of evidence (Wagenmakers Verhagen Ly Matzke Steingroever Rouder and Morey in press). Unlike null-hypothesis significance testing, Bayes factors have the advantage of distinguishing sensitive evidence for H0 from not much evidence at all. A *B* of above 3 indicates substantial evidence for the alternative hypothesis and below 1/3 substantial evidence for the null hypothesis. *B*s between 3 and 1/3 indicate data insensitivity in distinguishing null and alternative hypotheses (Dienes 2014; Jeffreys 1939). Here, *B*_H(0, *x*)_ refers to a Bayes factor in which the predictions of H1 were modelled as half-normal distribution with an SD of *x* (Dienes 2014); the half-normal can be used when a theory makes a directional prediction where *x* scales the size of effect that could be expected (so x can be chosen from e.g. relevant past studies). Kranick et al. (2013) provide an estimate of the sort of difference in intentional binding that could be found between different groups using conversion disorder patients; the difference between groups in tone binding was on the order of magnitude of about half the effect found in control participants. Bayes factors for group differences in each measure were therefore calculated using a half-normal distribution with SD based on half the total shift in the control group. The direction we used is that predicted by cue combination and sensorimotor pre-representation theories of intentional binding, given the assumptions discussed in the introduction (Wolpe and Rowe 2014). That is, the prediction was a greater outcome binding for meditators rather than controls, but a reduced action binding.

Bayes factors for solo action judgement group contrasts were calculated using a full-normal with SD = 80 ms based on the difference between meditators and non-meditators in the timing of an intention to move (Lush Naish and Dienes 2016). H1 for analysing differences between meditators and controls in standard deviations (SD) was specified using a uniform from 0 to the control groups’ SD, specified as B_U[0,*m*]_ where *m* is the maximum of the uniform distribution. A Bayes factor for the interaction of group and within-participant SD in baseline action and outcome trials was calculated using a uniform from 0 to the largest simple effect difference between groups (Dienes 2014).

## Results

**Fig. 1:**
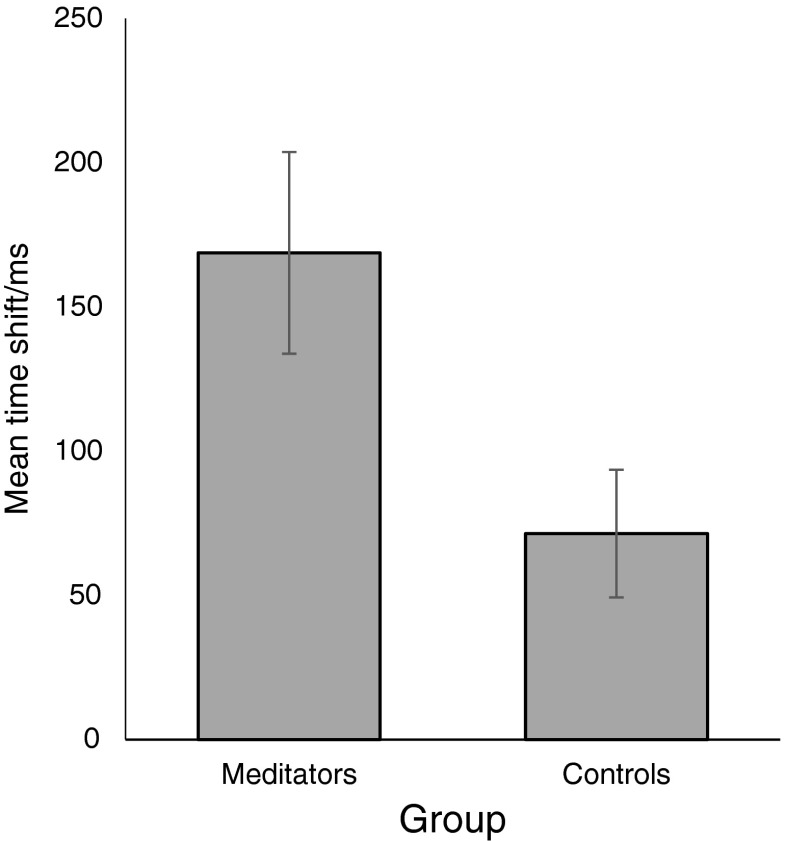
Total binding for meditators and age-matched controls. *Error bars* show standard errors

Figure 1 shows the overall intentional binding measures for each group. Meditators’ total binding (*M* = 168.8, *SD* = 98.9) was greater than that of age-matched non meditators (*M* = 71.7 ms, *SD* = 62.6), *t*(14) = 2.34, *p* = 0.034, *d* = 1.17, *B*_H(0, 40)=_ 3.95.

**Fig. 2:**
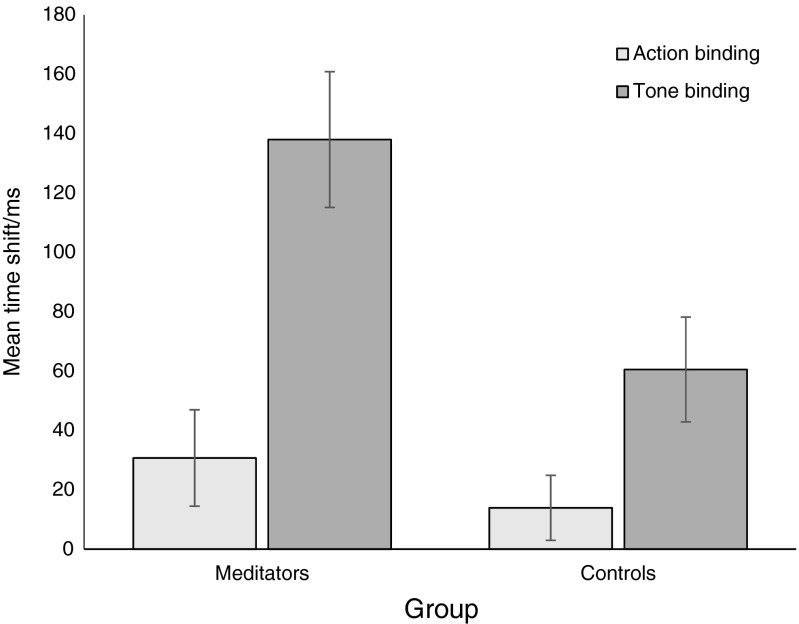
Action and outcome binding for meditators and age-matched controls. *Error bars* show standard errors

Figure 2 shows the action and outcome binding measures for each group. Meditators’ outcome binding (*M* = -138.02 ms, *SD* = 64.8) was greater than that of non-meditators (*M* = -60.5 ms, *SD* = 50.0), *t*(14) = 2.68, *p* = 0.018, *d* = 1.34, *B*_H(0, 32)=_ 6.57. There was no sensitive evidence for whether or not meditators’ action binding (*M* = 30.7 ms, *SD* = 45.9) was less than that of non-meditators (*M* = 13.9 ms, *SD* = 31.0), *t*(13) = 0.857, *p* = 0.406, *d* = 0.43, *B*_H(0, 9)=_ 0.80. For the prediction of action binding being greater for meditators rather than non-meditators, B_H(0, 9)_ = 1.20.

The raw simple effect of meditators’ within-participant SDs was 30.3 ms lower than that of controls for baseline action judgements and 11.2 ms lower for baseline tone judgements, so the raw interaction effect for within-participant SD on each measure between groups was 19.1 ms. There was no evidence one way or the other for whether or not there was an interaction, *F*(1, 14) = 0.427, *p* = 0.524, B_U[0, *30*]_ = 1.18.

## Discussion

We found that meditators showed greater subjective compression of the interval between an intentional action and its outcome than non-meditators (intentional binding). Specifically, meditators showed a greater shift in the perceived timing of an event toward the action that caused it; there was no sensitive evidence for a difference between meditators and non-meditators in the shift of action judgements toward its tone (Bayesian analysis showed these data to be insensitive in discriminating H1 from H0, so no firm conclusions can be drawn with regard to accepting the null hypothesis for action binding difference and this non-significant result will not discussed further). Meditators therefore reported a less veridical experience of timing of action outcomes than non-meditators. Furthermore, as intentional binding is considered an implicit measure of sense of agency, this result can be taken to show that meditators have a stronger sense of agency than non-meditators.

There may be different mechanisms underlying the two binding components, action binding and outcome binding (Wolpe Haggard Siebner and Rowe 2013). According to a predictive, sensorimotor model of outcome binding, the effect is due to a decreased latency in the perception of an outcome that results from the pre-representation of the predicted outcome of an action (Waszak et al. 2012). Therefore, an increase in outcome binding may be driven by stronger or more reliable predictive sensorimotor representations of the tone (Synofzik, Vosgerau and Voss 2013). Alternatively, when an outcome event is contingent upon an action event, the judgement of the time at which a particular event occurs may be a weighted average of each cue (Kawabe et al. 2013; Moore and Fletcher 2012). In this case, group differences in the timing shift of an outcome toward an action or of an action toward an outcome would reflect group differences in the relative weighting of each cue (on cue integration theory, we should expect lower variance of within-participant SD of action judgements than tone judgements; however, the evidence here was insensitive, so we can draw no conclusion as to how our results relate to this theory).

The increased outcome binding in meditators we report here could be attributable to differences in metacognition of motor intentions under both these accounts. Meditators may have more reliable or well specified sensorimotor representations of the predicted outcomes of an action, consistent with the claim that mindfulness involves close attention to concrete experience (Watkins 2015). Alternatively, on a cue combination approach meditators would show stronger outcome binding if improved metacognitive access to motor intentions or actions relative to external stimuli (like tones) affords higher precision of action timing relative to outcome timing. In this case, an increase in the weighting of the action cue in outcome judgements would lead to the timing judgement being drawn closer to the time of the action and therefore more outcome binding. In either case, greater accuracy of higher order mental states directed at intentions and their associated actions would, somewhat paradoxically, lead to a less veridical subjective experience of the timing of an intentional action’s outcome. The direct test of differences in precisions between groups for actions relative tones was insensitive, so this explanation is neither directly supported nor ruled out by the data.

Consistent with the suggestion that outcome binding is increased by improved metacognition of motor intentions, there is growing evidence that outcome binding is reduced when the contribution of intentions is reduced. A reduction in outcome binding has been shown to occur when participants incorrectly believed their action did not cause an outcome, a finding which may be attributable to the discounting motor intention related cues in judging the time of an outcome (Desantis Roussel and Waszak 2011). A post-hypnotically suggested experience of involuntariness has also been shown to reduce outcome binding (Lush et al., submitted). This finding may also be a result of the reduced contribution of motor intentions to the outcome judgement, as hypnotically induced experiences of involuntariness may be supported by the avoidance of motor intentions in forming metacognitive judgements of agency (Dienes 2012). Finally, activity in the pre-supplementary motor area (preSMA) reflects motor intentions (Lau Rogers Haggard and Passingham 2004). The disruption of activity in the preSMA by repetitive trans-cranial magnetic stimulation (rTMS) has been found to lead to a reduction in outcome binding (Moore et al. 2010) and the amplitude of the early readiness potential in the preSMA (associated with motor preparation) has been found to positively correlate with outcome binding (Jo et al. 2014).

Jo et al. (2014) reported a non-significant difference in intentional binding between mindfulness meditators and age-matched controls. However, Bayes factors can be employed to test evidence for the null hypothesis (Dienes 2014; Wagenmakers et al. in press; Dienes 2016), and in fact a meta-analysis of the results of the two studies provides sensitive evidence for an increase in overall binding and outcome binding (see Supplementary materials).

Mindfulness meditators report a slower subjective passing of time and are thought to experience an “extended now” (Wittmann and Schmidt 2014). However, here we report a shorter estimate of the time interval between an action and its outcome in meditators. These results can be taken to suggest that meditators have a stronger implicit, experiential sense of agency than non-meditators (Ryan and Rigby 2015). The study of group differences in action-outcome binding may therefore inform our understanding of the sense of agency and consequently disorders which involve distortions in the sense of agency, (e.g., functional motor disorders or schizophrenia). To this end, mindfulness meditators offer clear advantages over the use of the pharmacological interventions or clinical populations in which previous group differences in action-outcome binding have been observed. For example, the role of predictive cues in intentional binding has previously been studied by varying the probability of an outcome tone occurring (Moore and Haggard 2008). This method has been used to investigate intentional binding in schizophrenia and could be straightforwardly applied to investigate predictive contributions to outcome binding in meditators (Voss et al. 2010).

We report increased intentional binding, an objective measure of the sense of agency, in meditators over age-matched controls. Specifically, meditators showed a larger shift in the timing of an outcome toward the intentional action that caused it. While meditators here reported a less veridical experience of the timing of an event, we argue that this reflects that compared to non-meditators, mindfulness meditators have improved metacognition of motor intentions.
